# Few-Shot network intrusion detection based on prototypical capsule network with attention mechanism

**DOI:** 10.1371/journal.pone.0284632

**Published:** 2023-04-20

**Authors:** Handi Sun, Liang Wan, Mengying Liu, Bo Wang

**Affiliations:** State Key Laboratory of Public Big Data, College of Computer Science and Technology, Guizhou University, Guiyang, Guizhou, China; Technologico de Monterrey, MEXICO

## Abstract

Network intrusion detection plays a crucial role in ensuring network security by distinguishing malicious attacks from normal network traffic. However, imbalanced data affects the performance of intrusion detection system. This paper utilizes few-shot learning to solve the data imbalance problem caused by insufficient samples in network intrusion detection, and proposes a few-shot intrusion detection method based on prototypical capsule network with the attention mechanism. Our method is mainly divided into two parts, a temporal-spatial feature fusion method using capsules for feature extraction and a prototypical network classification method with attention and vote mechanisms. The experimental results demonstrate that our proposed model outperforms state-of-the-art methods on imbalanced datasets.

## 1. Introduction

Network intrusion detection is particularly important for network security, which ensures network security by distinguishing network attack traffic from normal network traffic [1]. Nowadays, deep learning (DL) as one of the most popular technologies has been applied to intrusion detection by many researchers. Deep learning-based intrusion detection is essentially a classification task that constructs a classification model by learning from a training set to identify network attack traffic. It has been shown in many studies that using DL can make intrusion detection models stable and high detection rate [2–6].

However, there is few instances of malicious traffic in the real network, so most intrusion detection datasets are imbalanced [7,8], that is, there is a significant class imbalance in the dataset. To address this, many researchers balance the dataset by increasing or decreasing the number of samples [9–11]. But as mentioned in [12], undersampling may lead to overfitting because of few samples, and it is difficult for oversampling to generate data that fit the real distribution.

To solve the above problem, we use Few-Shot Learning (FSL), which aims to achieve better classification performance using small amounts of labeled data [13]. The core of FSL is a similar process to human learning: it utilizes previous information to learn new tasks and requires not much data on new tasks. Research shows that FSL can make up for some shortcomings of DL, for instance, reducing the time spent collecting and labeling large datasets [14]. Therefore, FSL will be an effective method to solve the data imbalance problem caused by insufficient samples in network intrusion detection, and a scarcity of anomalous data is more realistic in real networks, FSL is becoming an alternative to traditional DL methods to simulate a more realistic environment.

This paper makes the key contributions to addressing the existing issues above, and is summarized as follows:

1. We propose a prototypical network classification model with attention and vote mechanisms. The spatial attention mechanism is used in the calculation of the class center to assign a weight to each region of the feature map and select more informative feature regions, to obtain more representative class centers. At the same time, when calculating the similarity between the sample and the class center, a voting mechanism is added to improve the few-shot classification ability.
2. We propose a capsule-based temporal-spatial feature fusion model for feature extraction. We replace neurons for pooling with capsules for less information loss in CNN. At the same time, we fuse the temporal-spatial features of traffic through the automatic feature extraction capability of DL to obtain a more representative feature representation and improve the detection effect of the model.
3. We improve the evaluation indicator by adding the proportion of few-shot categories in the calculation so that the evaluation results can better reflect the detection effect of small sample categories.

The rest of the paper is organized as follows: Section 2 presents the related work on techniques of the proposed method; Section 3 describes the proposed method in detail; Section 4 demonstrates the experiments to demonstrate the effectiveness of our method and compare it with state-of-the-art methods; Finally, Section 5 concludes this paper.

## 2. Related works

In this section, several issues related to the content of this paper are discussed, including network intrusion detection, FSL, and FSL in intrusion detection.

### 2.1 Network intrusion detection

In recent years, due to the rise of Artificial intelligence, researchers have applied DL to intrusion detection, which has made great achievements in detection efficiency and effect.

Zhang et al. [15] used the original data rather than statistical features, which reduced the loss of data information. In addition, they integrated an improved LeNet-5 and Long short term memory (LSTM) with better experimental results. Zhong et al. [16] proposed an intrusion detection framework that combines LSTM with AutoEncoder. They utilized AutoEncoder to calculate abnormal scores to flag network traffic and used these markers to train LSTM. The experimental results compared to Support Vector Machine (SVM), Isolation Forests (IF), and Gaussian Mixture Models (GMM). Li et al. [17] used the PCA algorithm to extract raw traffic features and proposed an intrusion detection model based on Transformer, which improves the detection ability of imbalanced datasets. Wei et al. [18] developed an attention-based LSTM model for more accurate detection. Lei et al. [19] leveraged multi-feature correlation for feature selection and applied the CNN with attention mechanism to capture features. Gupta et al. [20] suggested a cost-sensitive deep neural network, which assigns large weight to abnormal samples making it more costly of distinguishing the abnormal wrongly. Bedi et al. [21] proposed a two-layer ensemble structure for intrusion detection. The first layer integrates three structures for binary classification to identify attacks. These attacks are then sent to the second layer to be identified as different attack classes using multi-class eXtreme Gradient Boosting (XGBoost).

### 2.2 Few-shot learning

In the case of extremely limited training samples, FSL improves performance on new tasks with previous knowledge. FSL can be classified in three ways, data, model, or tuning algorithms [22].

The first type of FSL is mainly based on data enhancement. The sample size of FSL is usually small, so some data is generated from a small amount of original data to make the model perform well. A common method is to learn a generator by using the auxiliary labeled dataset. Andresini et al. [23] used generative adversarial network (GAN) for data enhancement. Their method leads to better detection accuracy when compared to other methods on four benchmark datasets. Although data augmentation is a straightforward approach, the resulting dataset is often task-specific and not easily extendable to other types of data, such as text or audio, which contain structural and grammatical information.

The second type of FSL is based on the model. The main models of FSL include siamese network [24], matching network [25], prototypical network [26], and relational network [27]. Zhang et al. [28] proposed a Contrastive learning-based Task Adaptation model (CTA) for few-shot intent recognition. They improve the prototypical network by changing the computing class prototype into the computing task prototype to improve the classification effect.

The third type of FSL is based on tuning algorithms. Xing et al. [29] brought dictionary learning methods into FSL and mapped feature embeddings to a more discriminative subspace for specific tasks. The experimental results show that the performance of the method has been improved.

### 2.3 Few-shot learning in intrusion detection

In intrusion detection, most methods are based on data enhancement and models, and few methods are based on tuning algorithms. Therefore, we mainly introduce the application of the first two kinds of methods in intrusion detection.

Iliyasu et al. [30] proposed a method for few-shot intrusion detection leveraging discriminative representation learning with a Supervised AutoEncoder. They first used known samples to train discriminant AutoEncoders for feature extraction and then used the AutoEncoder to fit a classification model with new attack categories in the stage of few-shot detection. Xu et al. [31] proposed a meta-learning framework to implement few-shot intrusion detection. This method defines a binary classification task, and constructs pairs of network traffic samples, including normal samples and malicious samples, for the model training. The experimental results show that the method is universal and performs well. Wang et al. [32] proposed a Siamese capsule network and an unsupervised subtype sampling scheme to solve the problem of insufficient training data of network attack traffic. Yu et al. [33] utilized the Siamese network as the classification model, consisting of two two-layer CNN. The method achieves an accuracy of 99.99% on the CIC-IDS-2017 and CIC-IDS-2018 datasets. To increase the amount of training data, Ye et al. [34] designed a pseudo sample generation algorithm called Latent Dirichlet Allocation (LDA). In real scenarios demonstrate, the experimental results show that the proposed method can effectively detect malicious traffic when only a few samples are learned. Wang et al. [35] ranked statistical features and used CNN to generate new features. The new features were combined with the original features and fed into a prototypical network for classification. Although this method achieved good detection results at the time, they used statistical feature data and the dataset used was not new enough.

## 3. The proposed method

**Fig 1:**
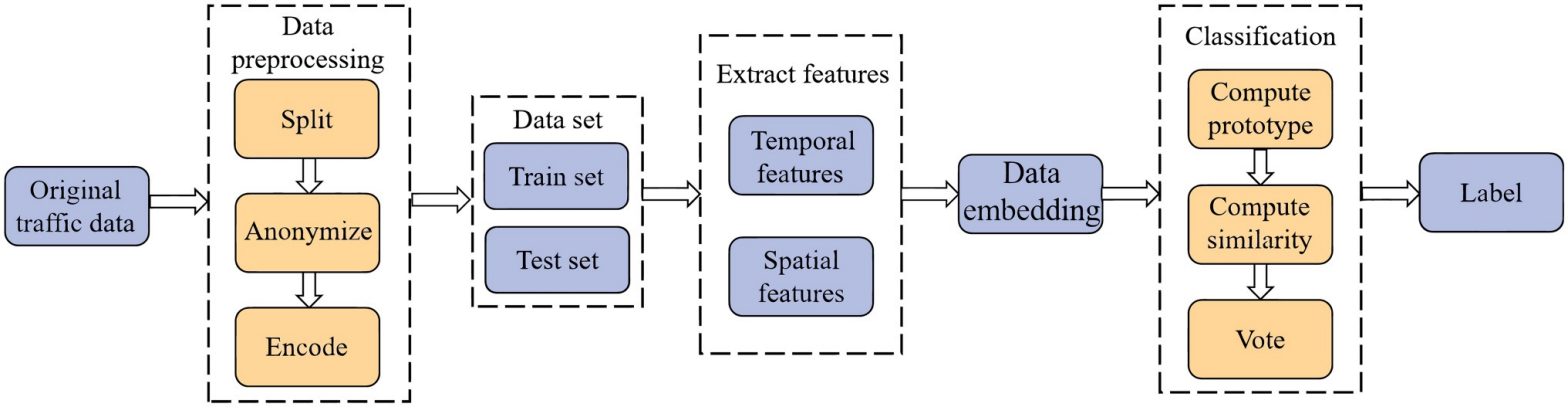
The architecture of the proposed method.

In this section, we design a few-shot intrusion detection method based on a prototypical capsule network with the attention mechanism according to reference [28]. Fig 1 is the architecture of our method. Before entering the data into the neural network, we preprocess the data to make it conform to the input format of the neural network. During the training and testing phase, respectively, we design a temporal-spatial feature fusion method using capsules for feature extraction and a prototypical network classification method with spatial attention and vote mechanisms. In this section, we describe each of these modules in detail.

### 3.1 Data preprocessing

**Fig 2:**
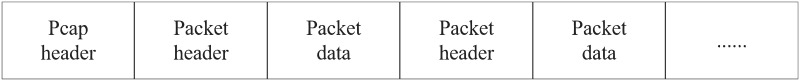
The structure of Pcap files.

This paper uses the dataset of Pcap files, CSE-CIC-IDS2018, instead of feature-ready CSV files. The structure of Pcap files is shown in Fig 2. The Pcap header contains file information, such as version number, timestamp, and file start flag. There are multiple packets after the Pcap header, each of them containing a packet header and packet data. However, the Pcap file cannot be directly input to the neural network, so we preprocess the data into the neural network input format.

**Fig 3:**
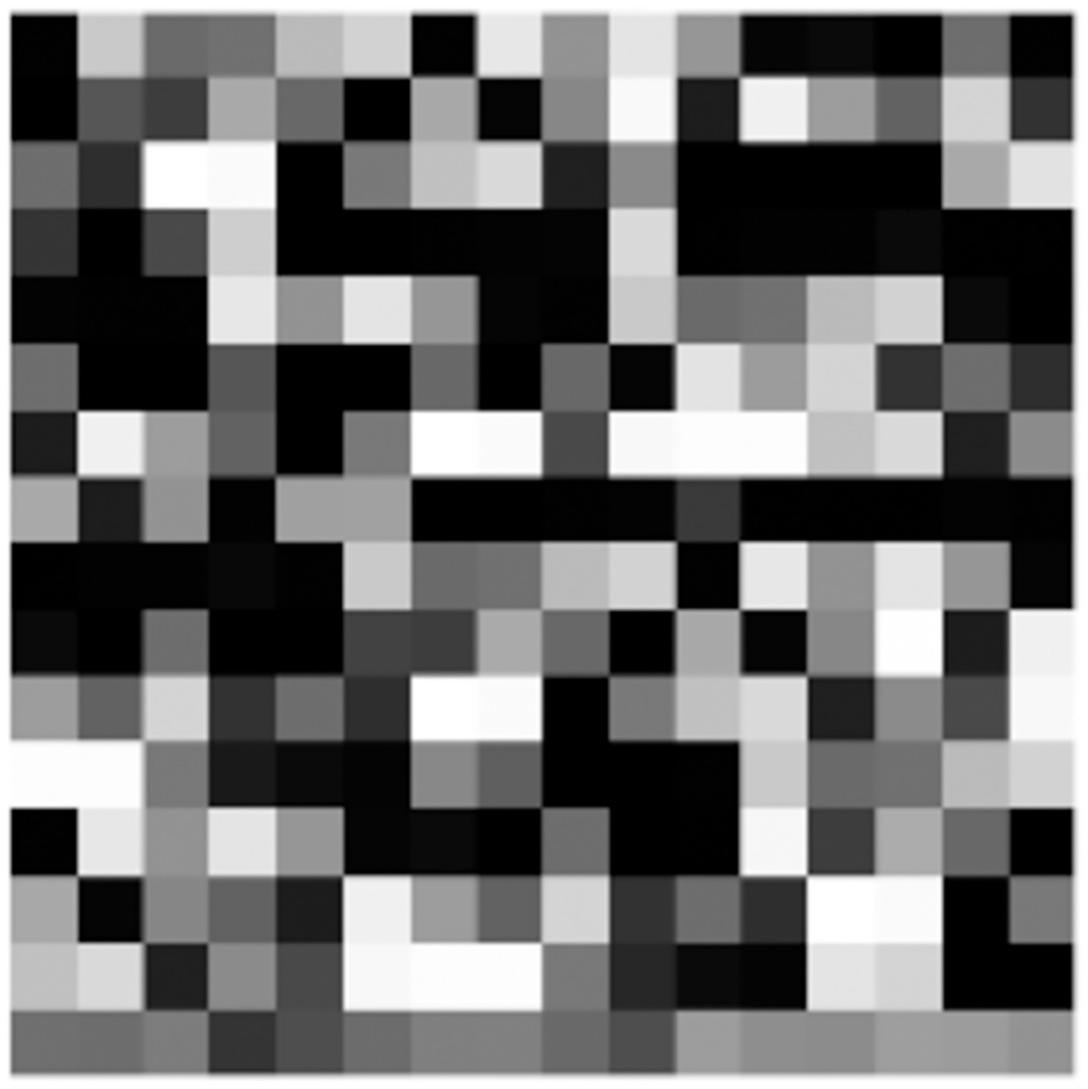
Visualization of a session sample.

**Fig 4:**
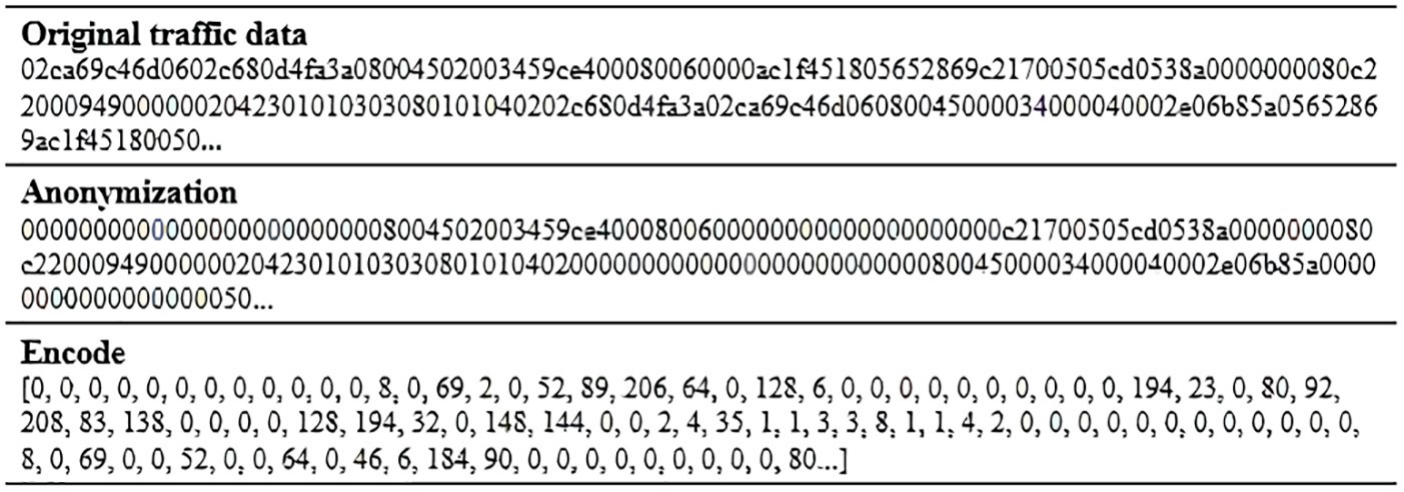
An instance of data preprocessing.

Network traffic granularity affects the analysis of data format and data distribution. Dainotti et al. [36] summarized five types of granularity commonly used in network traffic research, including TCP connections, flows, sessions, services, and hosts. We extract the quintuple (source IP address-source port, destination IP address-destination port, protocol) from the Pcap file, and integrate multiple packets into session samples according to the quintuple. Next, we anonymize addresses (both MAC addresses and IP addresses), because the model may classify sessions based on addresses only. There are two ways to anonymize, using random numbers of the same length or setting all addresses to the same, and we do the latter. We replace all MAC addresses with 00:00:00:00:00:00 and all IP addresses with 0.0.0.0. The session is byte data encoded in hexadecimal, so we need to convert the byte to numeric. A byte can represent a value of 0–255, which is consistent with pixel values in the image, so we convert a session to a pixel value, and the resulting image is shown in Fig 3. Fig 4 shows a session instance of split, anonymize, and encode. In addition, the neural network input size is limited, so the sample size *N* must be unified, and *N* will be determined experimentally later.

### 3.2 Temporal-spatial feature fusion method using capsules

**Fig 5:**
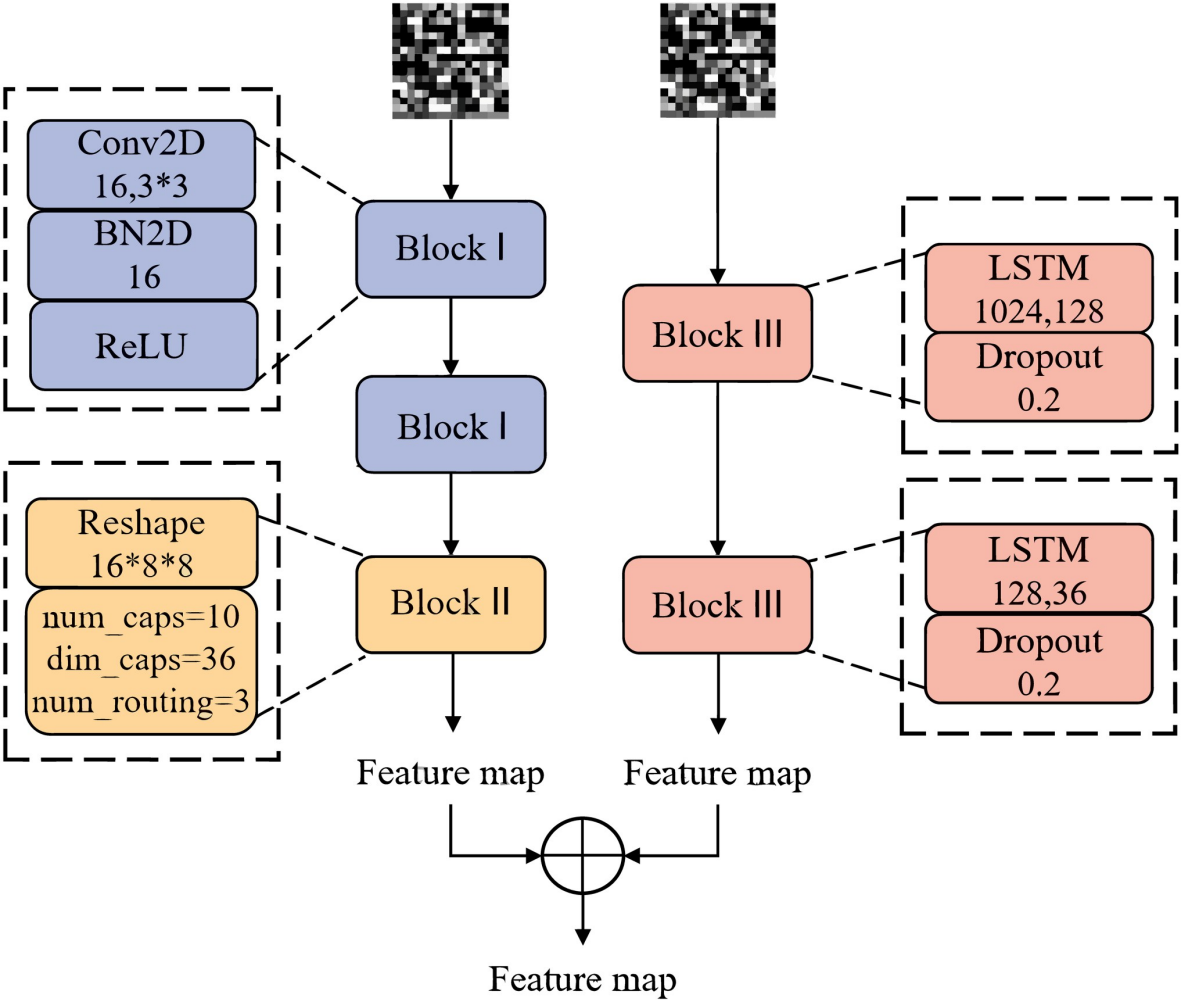
Temporal-spatial feature fusion model using capsule.

Network traffic has not only spatial characteristics, but also temporal characteristics, only one of them as the detection object is not comprehensive. Therefore, we design a temporal- spatial feature fusion model using capsules for feature extraction, and its structure shows in Fig 5.

#### 3.2.1 Spatial feature extraction

Although Convolutional neural networks (CNN) are often used to extract spatial features, there are certain limitations. First of all, data is transmitted between neurons as scalars. Since the scalar has only content and no direction, there are certain defects in the spatial position relationship between CNN identification features. While the feature location of network traffic is very sensitive, the confusion of location relationships will inevitably affect the accuracy of classification results. Secondly, there are max-pooling levels to explore the relationship between features in classical CNN, which will lead to the loss of high-level feature information extracted from the network. For few-shot data, both lack of samples and loss of feature information will undoubtedly affect the classification accuracy.

We utilize Capsule Networks (CapsNet) to extract spatial features of samples. Since network intrusion usually produces very significant local features, compared to other DL methods, CapsNet has the unique advantage of using local features. Meanwhile, dynamic routing of CapsNet is used to avoid feature loss caused by pooling operation. CapsNet is mainly composed of the main capsule layer and digital capsule layer. The operation process can be divided into three steps: the first step is matrix transformation, which is formulated as follows

$$u_{j}\left|i=W_{ij}u_{i}\right.$$ (1)

where *u*_*i*_ is the output of the low-level capsule, *W*_*ij*_ is the weight matrix between capsule layers, reflecting the spatial relationship between low-level features and high-level features. *u*_*j*_*|i* is the output of the high-level capsule predicted by the low-level capsule. The second step is input weighting, weighting and summing the prediction vector to get the output vector. The mathematical formula is as follows:

$$S_{j}=\sum_{i}c_{ij}u_{j}\left|i\right.$$ (2)

where the parameters are defined by a dynamic routing algorithm. The third step is nonlinear transformation. The capsule network uses a new activation function, called Squash function. The activation function formula can be expressed as

$$v_{j}=\frac{{\left\|s_{j}\right\|}^{2}}{1+{\left\|s_{j}\right\|}^{2}}\cdot \frac{s_{j}}{\left\|s_{j}\right\|}$$ (3)

**Fig 6:**
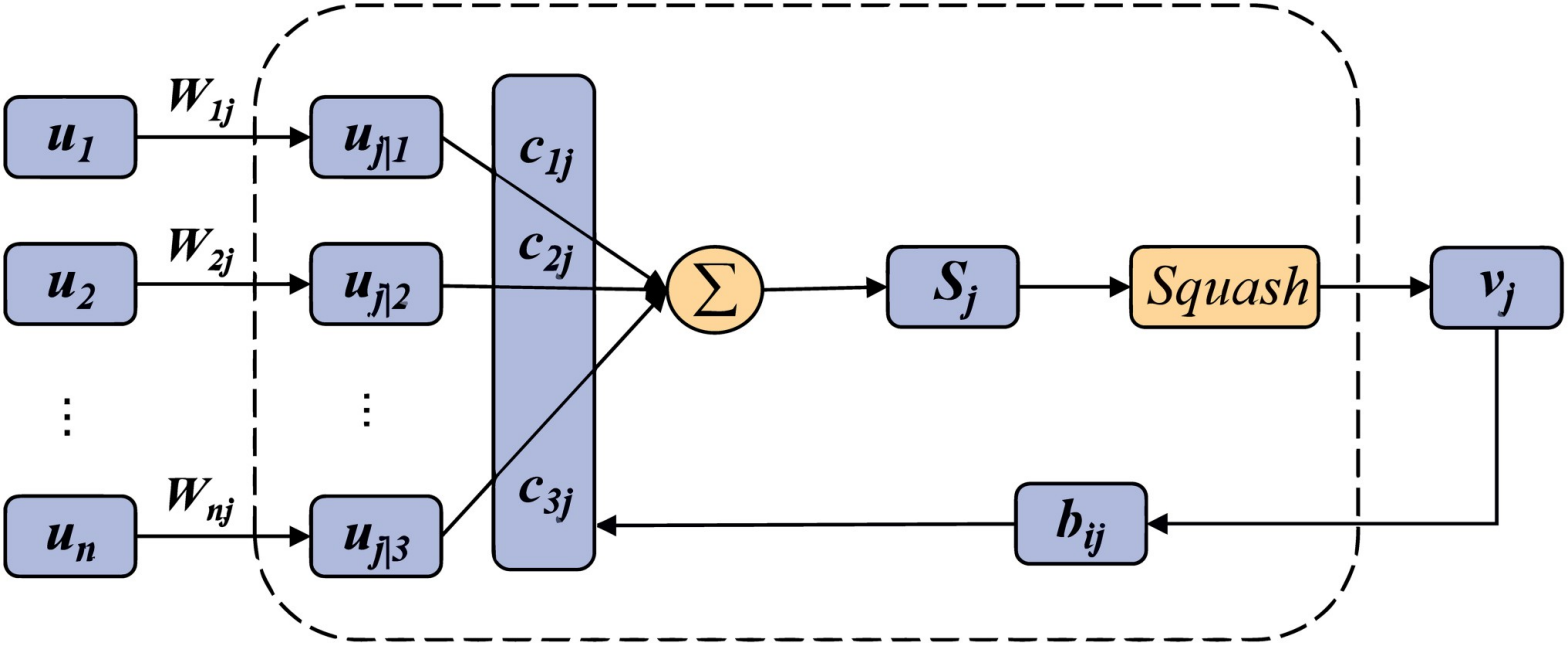
The calculation process of dynamic routing.

The calculation process of dynamic routing is shown in Fig 6. During the calculation, *b*_*ij*_ is initialized to 0, *c*_*ij*_ is obtained by calculating *b*_*ij*_ with the following formula:

$$c_{ij}=\frac{\text{exp}(b_{ij})}{\sum \;\text{exp}(b_{ij})}$$ (4)

*b*_*ij*_ is updated by the following formula:

$$b_{ij}=b_{ij}+v_{j}u_{j\left|i\right.}$$ (5)

The above process is cycled according to the number of dynamic routing iterations to finally obtain a set of optimal parameters.

#### 3.2.2 Temporal feature extraction

From the perspective of time characteristics, network traffic is a series of data packets that are consecutive in time. In this paper, LSTM is used to learn the temporal characteristics of samples. LSTM is a variant of Recurrent Neural Network (RNN), which is a good way to solve the gradient explosion and gradient disappearance problems of simple RNN. After the features of samples are generated by the two models respectively, the features are fused to the temporal-spatial features of samples and then passed into the classification model.

### 3.3 Prototypical network with attention and vote

**Fig 7:**
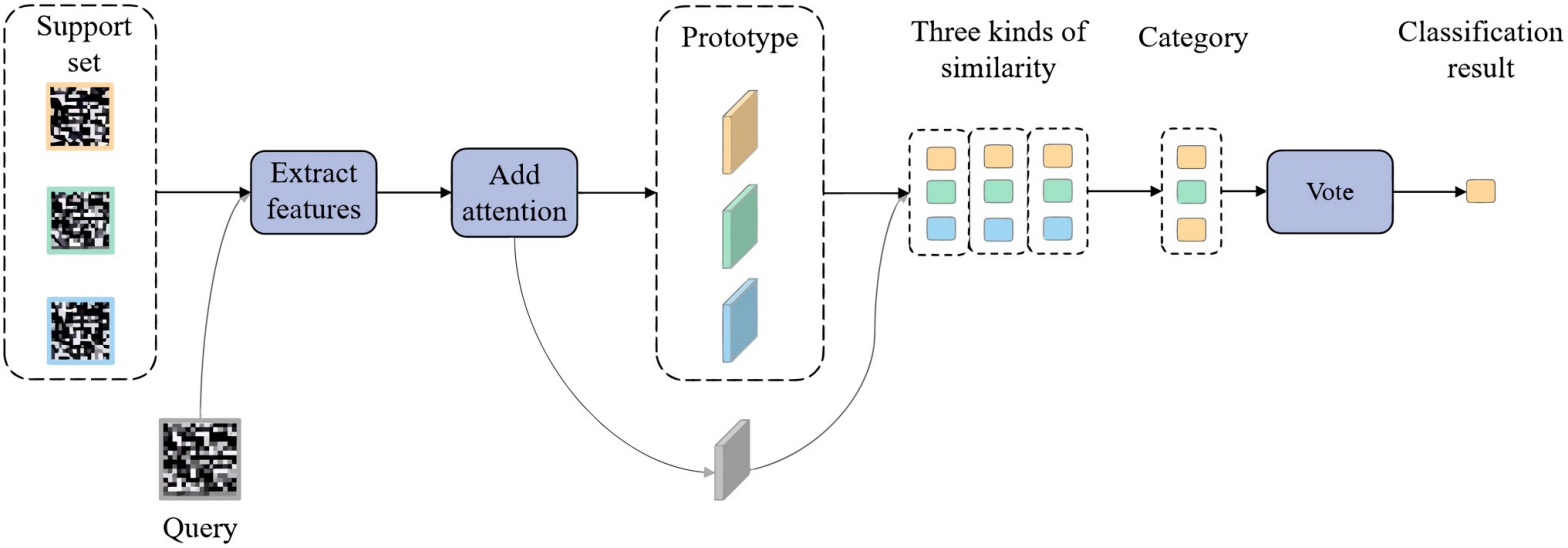
Proposed classification method.

In this paper, the prototypical network is employed as a classification model to accomplish the detection task. Its overall structure is shown in Fig 7. Based on the prototypical network, the spatial attention mechanism and the voting mechanism are added to improve performance. In this section, we introduce the proposed classification method from the perspective of two mechanisms.

#### 3.3.1 Calculate prototypes using attention

The basic task of network intrusion detection is to classify network traffic samples with a classifier. There are *K* samples and labels in task *D =* {(*x*_*1*_,*y*_*1*_),…(*x*_*i*_,*y*_*i*_)…(*x*_*k*_,*y*_*k*_)}, *x*_*i*_∈*R*^*h×w*^, *y*_*i*_∈{0,1,…*n*}. The purpose of this task is to build a classification model *f* whose input is the sample *x*_*i*_ and output is the predicted value of the corresponding label *y*_*i*_ of the sample. Generally in DL, the number of samples *K* is large and divided into train set and test set. We use few-shot learning, instead of focusing on a specific task, the model builds a task model *F*, and learns from the task in the task set T = {*T*_*A*_,*T*_*B*_,*T*_*C*_…}to complete a new task *T*_*N*_. The process of constructing a task set is as follows: in the first step, *N* classes are randomly selected from the dataset, and *2K* samples are randomly selected from each class. In the second step, from the N*2K samples extracted, *K* samples of each class are randomly selected as the train set of this task, and the remaining *K* samples of each class form the test set. In few-shot learning, the train set in each task is renamed the support set, and the test set is renamed the query set.

In this paper, we use the prototypical network. All extracted features of each class in the support set are summed and averaged to obtain the center of each class, which is the prototype. When a new sample is input into the network, the similarity between its features and all prototypes is calculated, and the maximum similarity is used to determine which category the sample belongs to.

**Fig 8:**
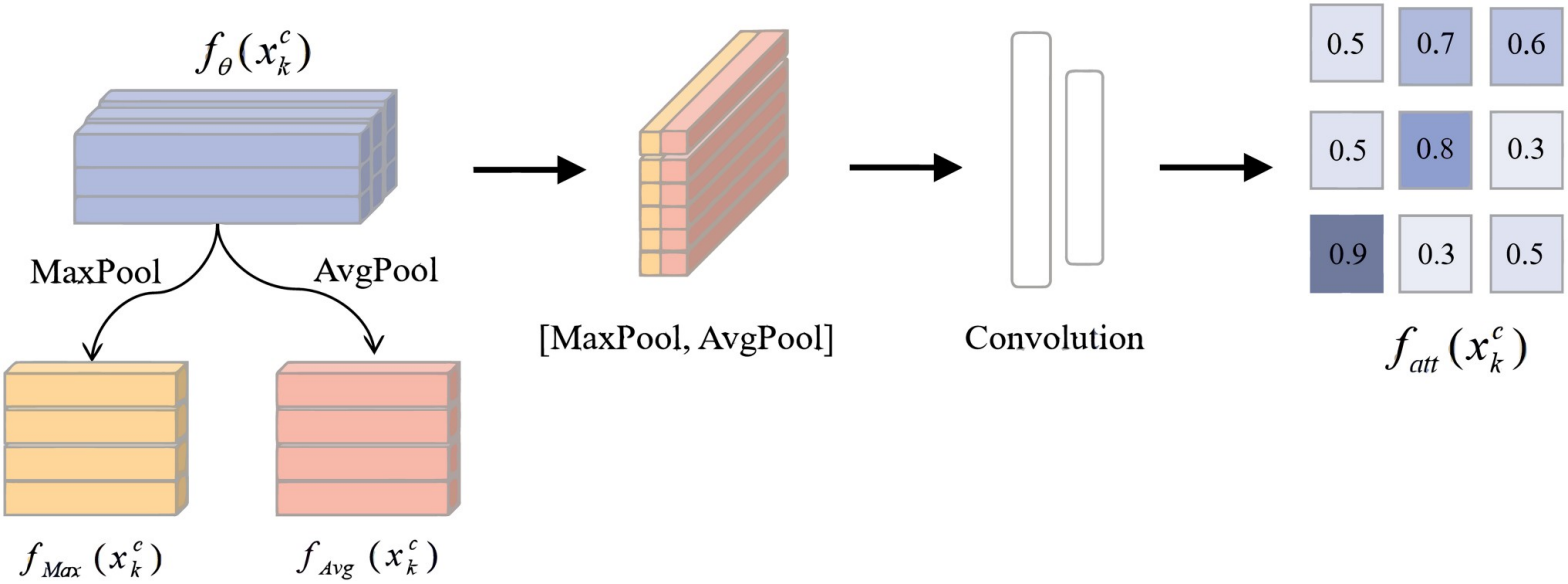
The process of spatial attention.

However, we believe that it is too simple to compute the prototype by arithmetic mean. The information content of different regions in the feature map is not the same, and the arithmetic mean cannot take advantage of this, which may lead to an inaccurate prototype, and affect the performance of the classification model. Therefore, We introduce spatial attention so that the model selectively outputs information, concentrating on key information and obtaining a more accurate prototype. Its process is unfolded in Fig 8. Suppose that sample $x_{k}^{c}$ (*k* = 1,…*K*, *c* = 1,…*C*) represents the *kth* sample selected from category *C*, and *f*_*θ*_ ($x_{k}^{c}$) is its feature map. We calculate the weight of each region in the feature map to obtain the attention map *f*_*att*_ ($x_{k}^{c}$) of the sample, the formula is as follows:

$$f_{att}(x_{k}^{c})=\sigma (f_{\phi }([MaxPool(f_{\theta }(x_{k}^{c}))\cdot AvgPool(f_{\theta }(x_{k}^{c}))]))$$ (6)

where average pooling and maximum pooling are carried out to get *f*_*Max*_ ($x_{k}^{c}$) and *f*_*Avg*_ ($x_{k}^{c}$). [.] represents the splicing operation, *σ* is the linear function Sigmoid. Then, the weighted feature map *f*_*ATT*_ ($x_{k}^{c}$) is obtained by multiplying *f*_*θ*_ ($x_{k}^{c}$) with *f*_*att*_ ($x_{k}^{c}$). In the training stage, the prototype C* of the class is calculated according to the formula (7).


$$C^{*}=\frac{1}{K}\sum_{k=1}^{K}f_{att}(x_{k}^{c})$$ (7)

#### 3.3.2 Vote classification

After computing the prototype, the samples of the query set are input into the model, and the similarity between their weighted feature maps and each prototype is calculated to complete the classification. To make the classification results more fault-tolerant, we not only use Euclidean distance but also cosine distance and Manhattan distance when calculating the similarity between samples and prototype. The formulas are shown in (8)–(10):

$$Dist(C^{*},f_{ATT}(x_{h}^{c}))=\sqrt{{\sum_{i=1}^{n}\left(C_{i}^{*}-f_{ATT}{\left(x_{h}^{c}\right)}_{i}\right)}^{2}}$$ (8)

$$\text{Cos}(C^{*},f_{ATT}(x_{h}^{c}))=\frac{\vec{C^{*}}\cdot \vec{f_{ATT}(x_{h}^{c})}}{\left\|C^{*}\right\|\times \left\|f_{ATT}(x_{h}^{c})\right\|}$$ (9)

$$Man(C^{*},f_{ATT}(x_{h}^{c}))=\sum_{i=1}^{n}\left|C_{i}^{*}-f_{ATT}{\left(x_{h}^{c}\right)}_{i}\right|$$ (10)

where the Euclidean distance measures linear distance, the cosine distance measures the directional difference, and the Manhattan distance calculates the total absolute wheelbases. We take the class corresponding to the minimum value of the three kinds of similarity as the classification result, then vote on the three classification results, and choose the classification result with more proportion as the final prediction result of the model.

## 4. Experiments

The experiments in this section have the following three objectives:

1. Choose the better values of the significant parameter: the length of session samples *N*.
2. Perform ablation experiments to verify the validity of the model.
3. Compare our method with the current related research works in terms of evaluation indicators, focusing on the detection effect of the few-shot class.

### 4.1 Dataset and evaluation indicators

In this paper, we selected CSE-CIC-IDS2018, a relatively new dataset in the field of intrusion detection. CSE-CIC-IDS2018 was generated by simulating the network traffic distribution of the Internet. It contains a large amount of original network traffic with extremely imbalanced attack data, makes it very suitable for our study. Considering the classification effect of few-shot classes in this paper, we select the categories with a small number of samples in the dataset and sample some categories with a large number of samples to form the dataset of this paper. The resulting dataset is shown in Table 1.

**Table 1:** The dataset of experiments.

Attack Types	Number of samples	Attack Types	Number of samples
Benign	25,040	Bruteforce-XSS	117
FTP-Bruteforce	2470	Bruteforce-web	138
DoS-Goldeneye	1105	Infiltration	114
Dos-Sloworis	625	SQL-Injection	241

To calculate the performance of the proposed method, evaluation indicators of Accuracy, Precision, Recall, and F1-Score are used as follows:

$$Accuracy=\frac{TP+TN}{TP+FP+FN+TN}$$ (11)

$$Precision=\frac{TP}{TP+FP}$$ (12)

$$Recall=\frac{TP}{TP+FN}$$ (13)

$$F1=\frac{2Recall\cdot Precision}{Recall+Precision}$$ (14)

where TP is the True Positive, FP is the False Positive, FN is the False Negative, and TN is the True Negative. Because of the imbalanced dataset, the Accuracy cannot reflect the true validity of our proposed model. Even if the model identifies malicious samples as normal samples, the Accuracy is still quite high due to the large proportion of normal samples. However, such high Accuracy is meaningless for intrusion detection, because few-shot attacks are identified as normal. In fact, the detection effect of these few-shot categories is very poor. Therefore, we use multiple evaluation indicators that more accurately respond to the effectiveness of the model.

To comprehensively measure all categories of the multi-classification task, calculate Macro-Averages (Macro-Avg) and Weighted-Averages (Weighted-Avg) for evaluation indicators. Macro-Avg is the arithmetic average, as shown in Eq (15):

$$Marco-Avg-P=\frac{1}{n}\sum_{i=1}^{1}P_{i}$$ (15)

Weighted-Avg uses the percentage of sample numbers as weights to reflect the detection effect of classes with large number of samples. However, this paper focuses on few-shot classes, so we improve the weight calculation process to increase the weights of few-shot classes. Taking Precision as an example, assume that there are *n* classes, *α*_*i*_ is the ratio between the number of samples of a class and the total number of samples, and *P*_*i*_ is Precision of a class. The formula is as follows:

$$Weighted-Avg-P=\sum_{i=1}^{n}\frac{1-\alpha_{i}}{\sum_{i=1}^{n}\left(1-\alpha_{i}\right)}P_{i}$$ (16)

### 4.2 Determine the length of session samples

During data preprocessing, we use padding or truncating to unify the length of session samples *N*. If *N* is too small, a large amount of payload information will be lost, while if *N* is too large, too many zeros will be filled, causing data noise. Whether *N* is too small or too large, the model performance will be reduced and the training effect will be low. Therefore, selecting the appropriate *N* can improve the representation ability and training performance of the model. In this section, we perform comparison experiments to determine the value of *N*. We set the candidate range to five values, *N* = {64, 144, 256, 1024, 2500, 4096}.

**Fig 9:**
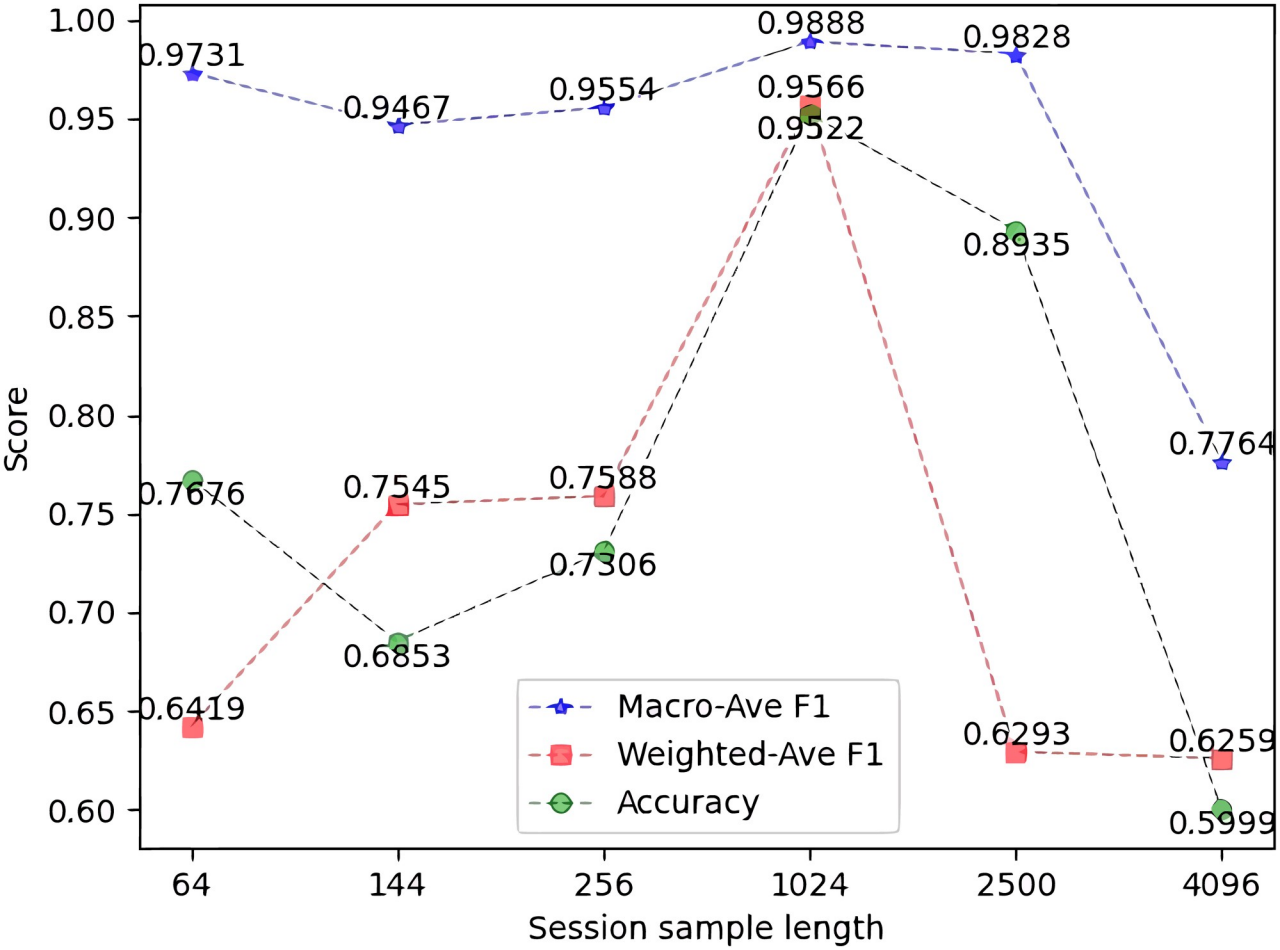
F1-score Macro-Ave, Weighted-Avg, and Accuracy of *N*.

The experiment training is on the train set, and the experimental evaluation is obtained on the test set. Except for the length of the samples, other parameters are the same in the experiment. *N* of the model with the relatively higher F1-score Macro-Ave, Weighted-Avg, and Accuracy is selected. The result is shown in Fig 9. The horizontal axis is the session sample length *N*, while the vertical axis is the above two values, which are represented by blue, red and green dashed lines, respectively.

It is observed in Fig 9 that, when *N* is 1024, the three values of the model are the highest, respectively 0.9522, 0.9566, and 0.9888. That is to say that when *N* is 1024, the detection performance of the model is the best. Therefore, we choose 1024 as the length of session samples.

### 4.3 Evaluate the proposed method

**Fig 10:**
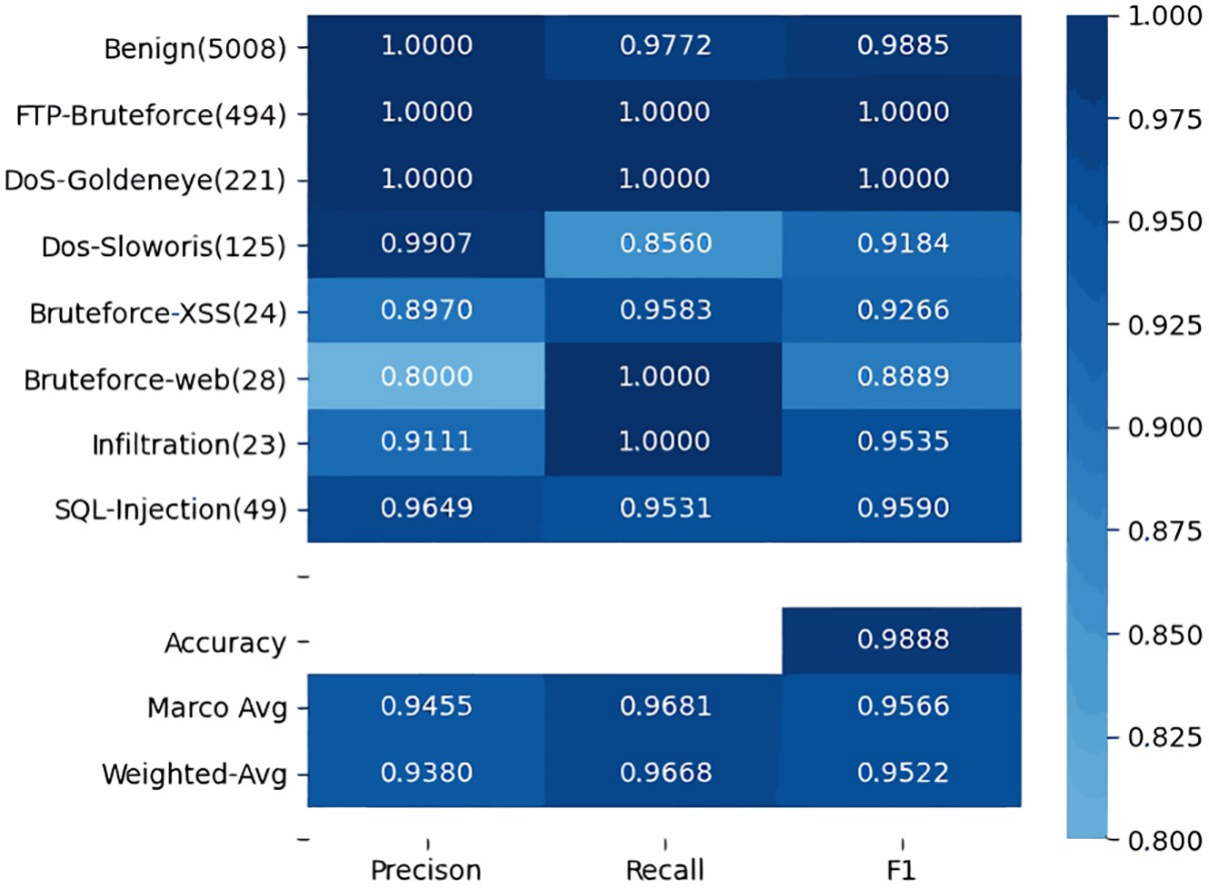
Heat map: The classification result of the proposed method.

As mentioned above, we select some categories from CSE-CIC-IDS2018 to form the experimental dataset. The length of session samples in the dataset is 1024, which is used as the input parameter of the model for training and testing. The result is shown in Fig 10 as a heat map.

Fig 10 represents the Precision, Recall, and F1-score for each category from benign to SQL-Injection. The darker the color, the higher the value, as shown in the right vertical bar. The value in parentheses indicates the number of session samples for that category in the test set. There are the overall accuracy, Macro-Ave and Weighted-Avg of precision, recall, and F1-score at the bottom. As is shown in Fig 10 that the proposed method performs well, which not only ensures the detection performance of most classes but also improves the detection effect of the few-shot classes. Therefore, the proposed method is effective for data imbalance in intrusion detection.

**Fig 11:**
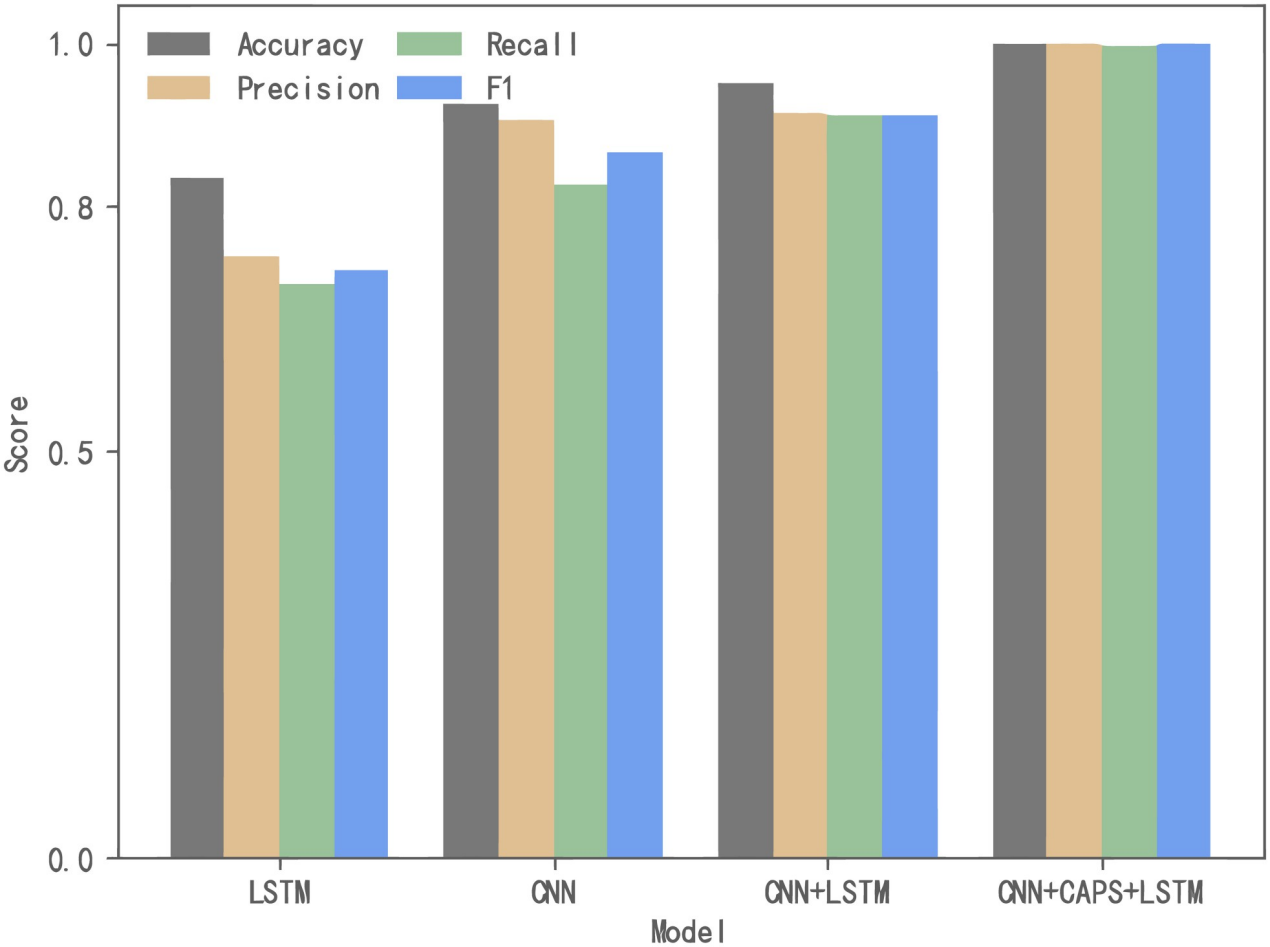
Evaluation of feature extraction model.

In addition to analyzing the overall results, we also evaluate the feature extraction model and the classification model separately to verify their performance. We use accuracy and Weighted-Avg of precision, recall, and F1-score as evaluation indicators. Fig 11 shows the ablation experiment results for the feature extraction model. It is observed that the scores of the four evaluation indicators are all the lowest when CNN or LSTM is used alone. After the fusion of CNN and LSTM, the Accuracy and Recall are improved by 3% and %9 compared to CNN with better performance used alone, indicating that our improvement is effective. On this basis, the capsule structure is added, and the four values were increased by about 5%, 3%, 6%, and 3%, to achieve the best detection effect.

**Fig 12:**
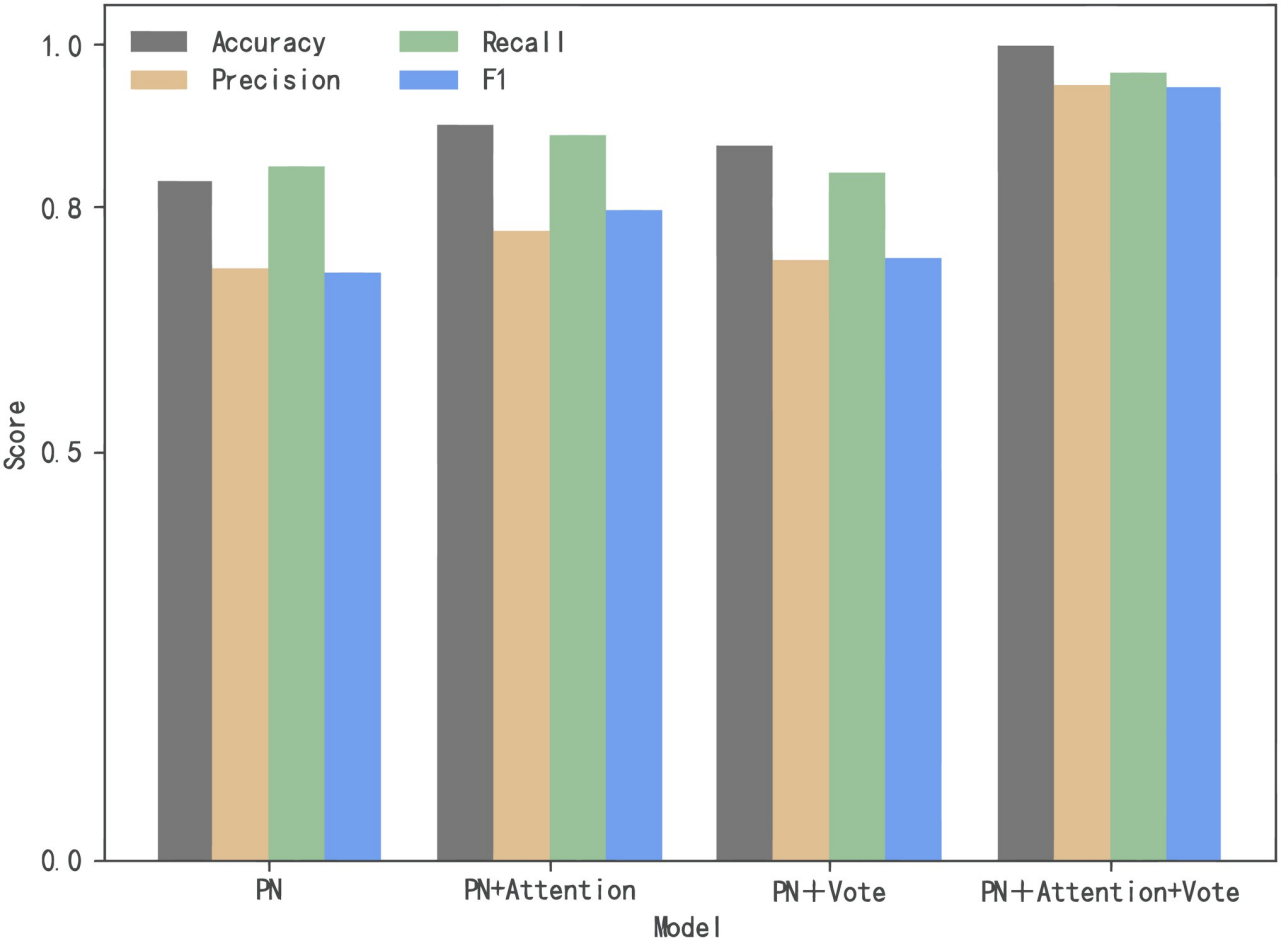
Evaluation of classification model.

Fig 12 shows the ablation experiment results for the classification model. The abscissa is the four contrasting models, which are prototypical network, prototypical network with attention mechanism, prototypical network with vote mechanism, and prototypical network with both attention and vote mechanism. As can be seen from Fig 12, compared with prototypical network alone, four indexes of the proposed model have been greatly improved, and the values are 0.9888, 0.9484, 0.9635, and 0.9456, respectively.

### 4.4 Comparison

In this section, we compare the proposed model with traditional machine learning methods and also with two few-shot intrusion detection methods for few-shot categories.

Table 2 shows the comparisons of the proposed model in terms of Weighted-Avg with five selected common traditional machine learning algorithms, namely K-Nearest Neighbors (KNN), Random Forest (RF), Support Vector Machine (SVM), XGBoost, Naive-Bayes (NB).

**Table 2:** Compared with traditional machine learning.

Algorithm	Weighted-Avg -P	Weighted-Avg-R	Weighted-Avg-F1
KNN	0.8427	0.8903	0.8659
RF	0.9157	0.9066	0.9111
SVM	0.9398	0.9530	0.9464
XGBoost	0.9106	0.9036	0.9071
Naive-Bayes	0.7512	0.8562	0.8003
Ours	0.9380	0.9668	0.9522

As mentioned above, this paper chooses to address the data imbalance problem in intrusion detection based on model-level instead of taking a data-level approach. Consequently, it is meaningless to choose a data-level method for comparison. We choose the same model-based improvement method, the PBCNN in reference [29]. At the same time, since this paper is inspired by reference [28] and improves on this basis, it needs to be compared with the Siamese capsule network proposed by reference [28] to prove the effectiveness of our improvement. On the same dataset, Precision, Recall, and F1 of few-shot classes, Bruteforce-XSS, Infiltration, Bruteforce-web, and SQL-Injection, are compared respectively. The results are shown in Tables 3 and 4.

**Table 3:** Compare with PBCNN.

Evaluation indicators	PBCNN			Ours		
	Precision	Recall	F1	Precision	Recall	F1
Bruteforce-XSS	0.2031	0.5417	0.2955	0.8970	0.9583	0.9266
Infiltration	0.8077	0.9130	0.8571	0.9111	1.0000	0.9535
Bruteforce-web	0.5000	0.1786	0.2632	0.8000	1.0000	0.8889
SQL-Injecton	0.5185	0.2857	0.3684	0.9649	0.9532	0.9590

**Table 4:** Compare with Siamese capsule network.

Evaluation indicators	Siamese capsule network			Ours		
	Precision	Recall	F1	Precision	Recall	F1
Bruteforce-XSS	0.6250	0.8333	0.7143	0.8970	0.9583	0.9266
Infiltration	0.6111	0.9565	0.7458	0.9111	1.0000	0.9535
Bruteforce-web	0.5778	0.9286	0.7123	0.8000	1.0000	0.8889
SQL-Injecton	0.7600	0.3878	0.5135	0.9649	0.9532	0.9590

It is shown in Table 3 that our method has a better detection effect on few-shot categories in the dataset. The four evaluation indexes are all higher than PBCNN, and the differences are very large. We believe that the number of samples in PBCNN dataset is larger than that used by us, and the detection effect is not as good as that shown in reference [29] when the dataset in this paper is used. While the Siamese capsule network performs better than PBCNN, it is still inferior to the proposed method. Since there is an unsupervised sampling operation in reference [28], however, this paper does not perform other operations on the data except for preprocessing.

To evaluate the time complexity, we calculate the training and testing time of the three models at the same condition. As shown in Table 5, our method spends a little more training time than the other two methods, because our method extracts not only spatial but also temporal features. And the testing time of the three methods is similar. In summary, our method gets a much better performance by compromising a small amount of time. It can be stated that our model can better adapt to the situation of data imbalance in intrusion detection, and the detection effect of few-shot categories is better.

**Table 5:** Comparison of time complexity.

	Training time (s)			Testing time (s)		
	Max.	Avg.	Min.	Max.	Avg.	Min.
PBCNN	1247.49	1201.39	1042.54	9.36	9.11	8.98
Siamese capsule network	1077.98	914.96	907.65	9.45	8.42	8.09
OURS	1686.63	1565.21	1553.02	9.93	9.33	9.12

## 5. Conclusions

In this paper, we point out the data imbalance problem caused by insufficient samples in network intrusion detection. We introduce few-shot learning to improve the detection model from both feature extraction and classification. In the feature extraction stage, we incorporate capsules in CNN and combine LSTM to extract the temporal-spatial features. In the classification stage, we improve the prototypical network of FSL models and introduce the spatial attention mechanism and the voting mechanism. After evaluation and comparison, the proposed method is proven to improve the detection performance of few-shot classes when the intrusion detection data is imbalanced.

In the future, we will design a multi-scale input model to make the payload information be utilized as much as possible, to compensate for the loss of payload information caused by unified input, and further improve the detection performance of few-shot classes.
